# Combining co-expression analysis and machine learning to explore the diagnostic value of complement and coagulation cascade-related genes in asthma and the functional role of SERPINB2

**DOI:** 10.1093/bfgp/elag007

**Published:** 2026-08-26

**Authors:** Jingjing Feng, Chengfang Xie, Weiduo Jin, Yanhong Huang, Yan Wang

**Affiliations:** Department of Respiratory Medicine, Shangyu People’s Hospital of Shaoxing, No. 517, Shimin Avenue, Shaoxing, Zhejiang, 312300, China; School of Medicine, Shaoxing University, No. 999 Zhongxing South Road, Shaoxing, Zhejiang, 312000, China; Department of Respiratory Medicine, Shangyu People’s Hospital of Shaoxing, No. 517, Shimin Avenue, Shaoxing, Zhejiang, 312300, China; School of Medicine, Shaoxing University, No. 999 Zhongxing South Road, Shaoxing, Zhejiang, 312000, China; Department of Respiratory Medicine, Shangyu People’s Hospital of Shaoxing, No. 517, Shimin Avenue, Shaoxing, Zhejiang, 312300, China; School of Medicine, Shaoxing University, No. 999 Zhongxing South Road, Shaoxing, Zhejiang, 312000, China; Department of Respiratory Medicine, Shangyu People’s Hospital of Shaoxing, No. 517, Shimin Avenue, Shaoxing, Zhejiang, 312300, China; School of Medicine, Shaoxing University, No. 999 Zhongxing South Road, Shaoxing, Zhejiang, 312000, China; Department of Neurosurgery, Nanjing Drum Tower Hospital, Affiliated Hospital of Medical School, Nanjing University, No. 321 Zhongshan Road, Nanjing, Jiangsu, 210008, China

**Keywords:** asthma, complement and coagulation cascades, WGCNA, machine learning, diagnosis

## Abstract

**Background:**

Optimizing diagnostic strategies for asthma is crucial for alleviating its high global disease burden. In recent years, the complement and coagulation cascades (CCC), as a central hub of innate immunity and inflammatory responses, has gained increasing attention for its association with asthma.

**Methods:**

Asthma-related datasets (GSE67472, GSE4302, and GSE76262) were downloaded from the Gene Expression Omnibus database. Subsequently, weighted gene co-expression network analysis and four machine learning algorithms—Decision Tree, K-Nearest Neighbors, Random Forest (RF), and Gradient Boosting Machine—were employed to identify key genes and construct a diagnostic model. SERPINB2 expression was then detected in clinical samples. The functional mechanisms of the key genes were validated using Transforming Growth Factor Beta 1 (TGF-β1)-treated Human Bronchial Epithelial Cells (BEAS-2B) cells through Quantitative Reverse Transcription Polymerase Chain Reaction (qRT-PCR), western blot, CCK-8 assay, and Co-IP experiments.

**Results:**

This research identified seven key genes: Serpin Family B Member 2 (SERPINB2), Complement C3 (C3), Complement C6 (C6), Colony Stimulating Factor 2 Receptor Subunit Beta (CSF2RB), Death Associated Protein Kinase 1 (DAPK1), Solute Carrier Family 16 Member 6 (SLC16A6), and CD69 Molecule (CD69). The diagnostic model constructed based on the RF algorithm exhibited robust discriminatory performance in both the training and validation sets. qRT-PCR assays on clinical tissue samples revealed significantly upregulated SERPINB2 expression in asthmatic tissues. *In vitro* experiments further revealed that SERPINB2 expression was upregulated in TGF-β1-treated BEAS-2B cells. Knockdown of SERPINB2 suppressed the expression of proteins related to epithelial−mesenchymal transition, inflammatory responses, and airway remodeling. Co-immunoprecipitation (Co-IP) assays indicated an association between SERPINB2 and C3 at the protein level.

**Conclusion:**

The RF model constructed based on seven CCC-related candidate genes exhibited promising diagnostic value for asthma. SERPINB2 may participate in airway epithelial inflammation and remodeling and may be associated with complement C3 at the protein level. These findings provide novel evidence for further elucidating the potential roles and clinical implications of the CCC cascade in the initiation and progression of asthma.

**Key points:**

- The random forest model constructed based on the screened features demonstrates robust and superior discriminative performance for asthma.
- Preliminary validation using clinical tissue samples confirmed the upregulated expression of SERPINB2 in asthma, which supports its potential diagnostic value.
- SERPINB2 mediates TGF-β1-induced airway epithelial inflammation and remodeling, and it interacts with complement C3 at the protein level.

## Introduction

Asthma is a chronic respiratory disease characterized by airway inflammation and remodeling, and it is triggered by complex genetic regulation and environmental allergen exposure [1]. Over 300 million people suffer from asthma globally, with its prevalence continuing to rise, resulting in ~1000 deaths daily [2]. The high incidence, mortality, and substantial economic burden of asthma constitute a major public health challenge worldwide [3]. Despite advancements in asthma management in recent years, precise diagnosis and phenotyping remain clinical challenges [4]. Traditional diagnostic indicators such as pulmonary function tests and eosinophil counts are valuable, but they are insufficient for comprehensively reflecting the molecular basis and individual heterogeneity of the disease [4–6]. Therefore, exploring highly specific biomarkers and constructing interpretable predictive models are of paramount importance for achieving early identification and personalized intervention in asthma.

The complement system and the coagulation system, as two core defense mechanisms of innate immunity and hemostasis, collectively form a rapid-response network against pathogens and tissue injury [7, 8]. Recent studies have revealed that the complement and coagulation systems can function as an integrated and synergistic entity through the highly interconnected “complement and coagulation cascades (CCC),” functioning as a critical nexus in inflammation and immune regulation. Their aberrant activation is closely related to the pathogenesis of chronic airway diseases such as asthma [9, 10]. Research indicates that complement components (e.g. C3 and C5) and their activation fragments (e.g. C3a and C5a) directly contribute to airway inflammation and hyperresponsiveness by modulating eosinophil infiltration, mast cell degranulation, and Th2-type immune responses [11–13]. Proteomic studies further demonstrate that complement C5 and its downstream components (C6–C9) are significantly upregulated in the sputum of patients with severe asthma, with expression levels positively correlating with type 2 inflammatory markers such as Fractional Exhaled Nitric Oxide (FeNO) and blood eosinophil counts [14, 15]. Concurrently, abnormal activation of the coagulation cascade has been confirmed to be closely linked to airway remodeling and small airway dysfunction [16, 17]. In summary, the complement and coagulation systems form a positive feedback loop during asthma pathogenesis, jointly amplifying airway inflammation and structural remodeling. Therefore, key molecules within the CCC may serve as potential biomarkers for asthma.

This study aims to systematically identify key genes associated with the CCC in asthma and to construct a diagnostic model by integrating Weighted Gene Co-expression Network Analysis (WGCNA) with multiple machine learning algorithms [Decision Tree (DTree), K-Nearest Neighbors (KNN), Random Forest (RF), and Gradient Boosting Machine (GBM)]. On this basis, we further preliminarily verified the expression profiles of hub genes using clinical bronchial tissue samples and explored their potential functions in processes associated with airway epithelial inflammation and remodeling *via in vitro* cellular experiments. The present study aims to provide evidence for the identification of asthma-related molecular biomarkers and the further dissection of the mechanisms underlying the CCC pathway.

## Materials and methods

### Data acquisition

The datasets GSE67472 and GSE4302, pertaining to the airway epithelium of asthma patients, were retrieved from the Gene Expression Omnibus (GEO) database (https://www.ncbi.nlm.nih.gov/geo/). The GSE67472 dataset, containing airway epithelium samples from 62 asthma patients and 43 healthy controls, served as the training set. The GSE4302 dataset was used as an independent validation set, which included samples from 42 untreated asthma patients, 28 healthy controls, 19 asthma patients treated with Flovent, and 13 asthma patients receiving placebo treatment. For external validation of the model, only the 42 untreated asthma patients and 28 healthy controls from the GSE4302 dataset were included, yielding a total of 70 samples to eliminate the confounding effects of therapeutic interventions on gene expression profiles and model performance evaluations. The 32 asthma patient samples receiving Flovent or placebo treatment were excluded from model validation and were solely utilized for subsequent analyses of candidate gene therapeutic responses. All expression data underwent log2 transformation and were normalized using the Robust Multi-array Average (RMA) algorithm. Furthermore, the dataset GSE76262, comprising induced sputum samples from 25 moderate and 93 severe asthma patients, was included to evaluate the correlation between the expression levels of key genes and asthma severity.

Gene sets related to the CCC pathway were retrieved from the Kyoto Encyclopedia of Genes and Genomes (KEGG) database (https://www.kegg.jp/pathway/hsa04610), encompassing a total of 88 genes (Supplementary Table S1).

### WGCNA

First, the coefficient of variation (CV) for each gene was calculated, and genes with a CV ≥ 4% were retained to filter out those with relatively low or stable expression levels. Subsequently, the “GSVA” R package [18] was employed to calculate the CCC activity score for each sample. Following this, a co-expression network was constructed based on the filtered gene expression matrix using the “WGCNA” R package [19]. The specific steps were as follows: (i) Sample clustering analysis was performed to exclude outlier samples significantly deviating from the majority, with a height cut threshold set at 5e+6. (ii) An appropriate soft thresholding power was selected to construct a weighted adjacency matrix adhering to a scale-free topology, which was then transformed into a topological overlap matrix (TOM) to minimize the impact of noise on network construction. (iii) Hierarchical clustering analysis was performed on the genes, setting the minimum module size to 30 genes, and modules with a similarity >0.25 were merged. Finally, modules with an absolute correlation value >0.5 to the CCC activity score were identified as key modules for subsequent analysis.

The online tool GEO2R (https://www.ncbi.nlm.nih.gov/geo/geo2r) was utilized to identify differentially expressed genes (DEGs) between the disease and control groups, using the thresholds of |log₂FC| > 0.585 and False Discovery Rate (FDR) < 0.05. Gene identifiers were then converted to standard gene symbols using the “org.Hs.eg.db R package” [20], and non-protein-coding genes were filtered out. Subsequently, two core gene sets for in-depth analysis were obtained by intersecting: the DEGs with CCC-related genes, and the DEGs with genes from the WGCNA-identified modules. A volcano plot of the DEGs was generated to visually illustrate their expression distributions.

### Model construction

To address the class imbalance between the asthma samples (*n* = 62) and healthy control samples (*n* = 43) in the training data, a combined approach of Synthetic Minority Oversampling Technique (SMOTE) and random undersampling was applied with the “smotefamily” R package (https://CRAN.R-project.org/package=smotefamily). Specifically, the mean sample size of the two classes was set as the target number. For the majority class, random sampling was used to reduce its size to the target number; for the minority class, synthetic samples were generated *via* the SMOTE algorithm to reach the target number. Ultimately, a balanced dataset comprising 53 asthma samples and 53 healthy controls was obtained. Subsequently, four machine learning models were constructed using the “caret” R package [21] for predicting asthma risk: DTree, KNN, RF, and GBM.

During model training, a hold-out method was employed in conjunction with five-fold cross-validation repeated five times (repeats = 5) and Grid Search (GridSearchCV) to optimize the hyperparameters for each model. The specific tuning ranges were as follows: DTree: The complexity parameter (cp) was tuned within a range of 0.01−0.03 with a step size of 0.005, while the minimum samples for a split (minsplit) and the maximum depth (maxdepth) were fixed at 30. KNN: The number of neighbors (k) was searched from 3 to 21 with a step size of 2. RF: The number of features randomly sampled at each split (mtry) was optimized from 2 to 8 with a step size of 1, and the number of trees (ntree) was set to 300. GBM: A combination of four parameters was searched: the number of trees (n.trees: 5, 10, and 20), interaction depth (interaction.depth: 1, 3, and 5), shrinkage (shrinkage: 0.01 and 0.1), and the minimum number of observations in a node (n.minobsinnode: 5, 10, and 20).

### Model performance evaluation and correlation analysis

The diagnostic and generalization capabilities of the constructed machine learning models were evaluated using both the training and validation sets. First, a confusion matrix was employed to visualize the classification results, based on which key performance metrics, including accuracy, F1-score, specificity, and sensitivity, were calculated to quantify the model’s classification efficacy. Accuracy: This metric quantifies the overall proportion of correct classifications made by the model, calculated as (TP + TN)/(TP + TN + FP + FN). Precision and recall: Recall, also known as sensitivity, is calculated as TP/(TP + FN). It quantifies the model’s ability to identify all positive instances. Additionally, receiver operating characteristic (ROC) curves were plotted, and the area under the curve (AUC) was computed to comprehensively evaluate the classification performance of each model.

To enhance the model interpretability and clinical translational potential, the SHAP (Shapley Additive Explanations) method implemented in the “shapviz” R package [22] was used to analyze the contribution of each feature to the model’s predictions. By calculating and visualizing the SHAP values of key features, genes with a significant impact on the prediction outcome were identified. A higher gene expression level related to a larger positive SHAP value indicates a greater contribution to the model’s prediction of the asthma class. Furthermore, literature searches were conducted to validate the known associations of these genes with the disease, thereby supporting the biological plausibility of the model.

To assess the network correlations between candidate genes and the CCC pathway, Pearson correlation analyses were performed on the 7 candidate genes and the 80 detectable genes among the 88 CCC-related genes within the GSE67472 training cohort. The Benjamini–Hochberg (BH) method was applied for multiple testing correction, and an adjusted *P* < .05 was defined as statistically significant.

### Collection of clinical samples

A total of 10 lobar or segmental bronchial tissue specimens were collected in this study, including five asthma patients and five non-asthmatic controls. Tissue samples were obtained from all subjects during examinations or surgical procedures at Shangyu People’s Hospital of Shaoxing (Approval No.: 2026-021-01). This study was approved by the Shangyu People’s Hospital of Shaoxing Ethics Committee, and written informed consent was acquired from all participants.

Inclusion criteria for the asthma group: (i) aged 18–75 years with no restriction on gender; (ii) consistent with the diagnostic criteria of a positive bronchodilator test [an increase in forced expiratory volume in 1 s (FEV1) ≥ 12% with an absolute elevation ≥ 200 ml] or a positive bronchial provocation test (PC20 < 8 mg/ml); (iii) no acute asthma exacerbations within the preceding 4 weeks; and (iv) no prior treatment with biological agents. Exclusion criteria: (i) complicated with other chronic respiratory diseases such as chronic obstructive pulmonary disease and bronchiectasis; (ii) respiratory tract infection occurring within the previous 4 weeks; and (iii) concurrent autoimmune diseases, coagulation disorders, or ongoing anticoagulant therapy. Non-asthmatic control subjects were patients who underwent lobectomy or segmentectomy for non-asthma-related diseases during the same period. Bronchial tissues located at a certain distance from the lesion sites and pathologically confirmed to be without obvious abnormalities were harvested. Controls had no history of allergic diseases, including asthma, allergic rhinitis, and atopic dermatitis, and were free of chronic obstructive pulmonary disease, bronchiectasis, and recent respiratory tract infections. All collected tissue specimens were immediately preserved in liquid nitrogen and subsequently stored at −80°C for subsequent RNA quantification.

### Cell culture and treatment

Human bronchial epithelial BEAS-2B cells were sourced from Icellbioscience Biotechnology Co., Ltd (Shanghai, China). The BEAS-2B cells were cultured in RPMI-1640 medium supplemented with 10% fetal bovine serum (10099141C, Thermo Fisher Scientific, USA) at 37°C with 5% CO₂. Given that Transforming Growth Factor Beta 1 (TGF-β1) acts as a critical mediator driving airway EMT and structural remodeling, BEAS-2B cells were treated with 10 ng/ml TGF-β1 (Thermo Fisher Scientific) for 48 h to establish an *in vitro* model of airway epithelial remodeling [23]. This model was applied to mimic asthma-associated EMT and remodeling processes, rather than fully recapitulating the Th2 inflammatory microenvironment driven by IL-4, IL-5, and IL-13. Untreated cells served as controls.

Small interfering RNA (siRNA) targeting SERPINB2 (si-SERPINB2) and its negative control (siNC) were designed and synthesized by Sangon Biotech (Shanghai, China). We performed transfection experiments with Lipofectamine 3000 reagent (L3000150, Invitrogen, USA).

### Quantitative Reverse Transcription Polymerase Chain Reaction (qRT-PCR)

We extracted RNA from BEAS-2B cells or clinical samples with Trizol reagent (15596018CN, Invitrogen, USA). RNA was reverse-transcribed with the SuperScript™ III kit (18080051, Invitrogen, USA). qPT-PCR was conducted with SYBR Premix Ex Taq II (4385610, Thermo Fisher Scientific, USA) on the ABI 7500 platform. Gene expression was quantified and normalized to Glyceraldehyde-3-Phosphate Dehydrogenase (GAPDH) using the 2^−ΔΔCt^ method. The primer sequences are as shown in Table 1.

**Table 1:** Primer sequences for qRT-PCR.

Gene	Sequence	
SERPINB2	Forward	5'-ATAACCAGAGAACAACCAGATTGAA-3'
	Reverse	5'-ATGGCCATGGTGGACGAGAT-3'
IL-1β	Forward	5'-AACCTCTTCGAGGCACAAGG-3'
	Reverse	5'-GGCGAGCTCAGGTACTTCTG-3'
IL-6	Forward	5'-GGAGCAGTGGCTTCGTTTC-3'
	Reverse	5'-GAGGATGGCTGGATGGTTTCA-3'
TSLP	Forward	5'-GGACTGGCAATGAGAGGCAA-3'
	Reverse	5'-GGGAACATACGTGGACACCC-3'
GAPDH	Forward	5'-AATGGGCAGCCGTTAGGAAA-3'
	Reverse	5'-GCGCCCAATACGACCAAATC-3'

### Western blot

Proteins were extracted from the cells using Radioimmunoprecipitation Assay (RIPA) buffer (20–188, Sigma Aldrich, MO, USA), separated by 10% Sodium Dodecyl Sulfate–Polyacrylamide Gel Electrophoresis (SDS-PAGE), and subsequently transferred onto Polyvinylidene Fluoride (PVDF) membranes (Millipore, MA, USA). The membranes were blocked with 5% skimmed milk for 2 h, with overnight incubation at 4°C with corresponding primary antibodies. After washing, the membranes were incubated with Horseradish Peroxidase (HRP)-conjugated goat anti-rabbit IgG secondary antibody (ab6721, 1:1000) for 2 h. The relative band intensity was quantified using ImageJ software. Data were normalized to the internal reference GAPDH. Information for all primary antibodies, purchased from Abcam (Cambridge, UK), is as follows: SERPINB2 (ab267463, 1:1000); C3 (ab200999, 1:500); E-cadherin (ab227639, 1:1000); N-cadherin (ab76011, 1:2000); vimentin (ab137321, 1:1000); Alpha-Smooth Muscle Actin (α-SMA) (ab124964, 1:1000); collagen I (ab138492, 1:1000); fibronectin-1 (ab268020, 1:1000); and GAPDH (ab9485, 1:2500).

### Cell viability assay

Cell viability was determined by a Cell Counting Kit-8 (CCK-8, C0037, Beyotime, Shanghai, China). BEAS-2B cells underwent TGF-β1 treatment and siRNA (siNC or si-SERPINB2) transfection. To each well, 10 μL of CCK-8 solution was added. Absorbance was read at 450 nm with a microplate reader.

### Co-immunoprecipitation (Co-IP) assay

Following cell lysis, the BEAS-2B cell lysates were pre-cleared by incubation with IgG and Protein A + G agarose beads (P2089, Beyotime, Shanghai, China) for 2 h. The pre-cleared lysates were then collected and incubated overnight at 4°C with a primary antibody. Subsequently, Protein A/G agarose beads were added to the mixture to isolate the antibody-bound protein complexes. The immunoprecipitated proteins were eluted by boiling in loading buffer at 100°C for 20 min and subsequently analyzed by SDS-PAGE and immunoblotting. Band intensities were quantified using ImageJ software.

### Enzyme-linked immunosorbent assay

Enzyme-linked immunosorbent assay was performed to detect the expression levels of IL-1β, IL-6, and Thymic Stromal Lymphopoietin (TSLP) in the culture supernatants of BEAS-2B cells from each group. BEAS-2B cells were divided into four groups: Control group, TGF-β1 group, TGF-β1 + si-NC group, and TGF-β1 + si-SERPINB2 group. After treatment, cell supernatants were collected and centrifuged to remove cellular debris. The concentrations of IL-1β, IL-6, and TSLP were measured using human IL-1β ELISA Kit (PI305, Beyotime, Shanghai, China), human IL-6 ELISA Kit (PI330, Beyotime, Shanghai, China), and human TSLP ELISA Kit (PT894, Beyotime, Shanghai, China), respectively. All experimental procedures were strictly conducted in accordance with the manufacturer’s instructions. The absorbance value was detected at 450 nm using a microplate reader, and the concentrations of inflammatory cytokines were calculated based on standard curves. All experiments were independently repeated at least three times.

### Statistical analysis

Bioinformatics analyses were mainly implemented using R software. The Wilcoxon rank-sum test was adopted to compare intergroup differences in gene expression. Spearman’s rank correlation analysis was used to assess correlations among candidate genes, while Pearson correlation analysis was applied to evaluate correlations between candidate genes and CCC pathway genes; the BH method was utilized for multiple testing correction. ROC curves and AUC were generated to evaluate the discriminatory performance of the model and single genes. Based on the confusion matrix, accuracy, precision, recall, F1 score, sensitivity, and specificity were further calculated.

Statistical analyses for wet laboratory experiments were performed with GraphPad Prism. All cellular experiments including qRT-PCR, CCK-8, and ELISA were independently repeated no less than three times, and data were presented as the mean ± SEM. Two-tailed Student’s *t-*test was used for comparisons between two groups, whereas a one-way ANOVA followed by corresponding *post hoc* tests was applied for multiple group comparisons. Each subject was treated as an independent observation for the statistical analysis of clinical samples. Western blot and Co-IP results were only displayed qualitatively without statistical inference based on biological replicates. All statistical tests were two-sided, and a *P* < .05 was defined as statistically significant.

## Results

### Construction of weighted gene co-expression network

**Figure 1:**
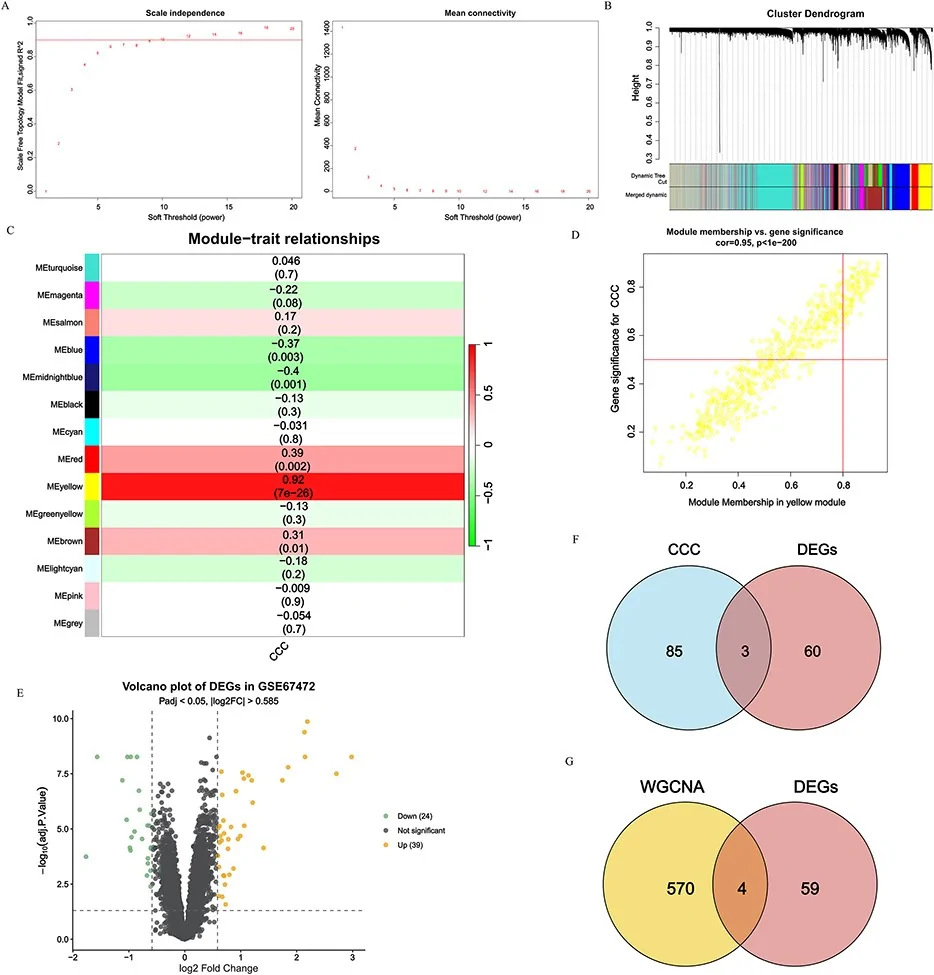
WGCNA. (A) Signed scale-free topology fitting index (signed *R*^2^, left) and mean connectivity (right) under different soft-thresholding powers. (B) Clustering dendrogram of the WGCNA network. (C) Heatmap showing the correlation between module eigengenes and the CCC score. (D) Scatter plot of GS for CCC versus MM in the yellow module. (E) Volcano plot illustrating the 63 DEGs between normal and asthmatic groups in the GSE67472 dataset, of which 39 were upregulated and 24 were downregulated. (F, G) Venn diagrams showing the intersection of DEGs with CCC-related genes (F) and DEGs with the yellow module genes (G).

To precisely identify key genes associated with the CCC, a WGCNA was performed. The gene co-expression network was constructed by setting the soft-thresholding power *β* = 10 (signed *R*^2^ = 0.90) (Fig. 1A). Subsequently, a phylogenetic tree and a heatmap were used to quantify module similarity based on correlation. The WGCNA analysis revealed a total of 14 modules (Fig. 1B and C). Among them, the yellow module showed the strongest positive correlation with the CCC score (correlation = 0.92, *P* < .0001). Based on a threshold of gene significance (GS) >0.5 and module membership (MM) >0.8 within the yellow module, 536 candidate genes highly connected in this module were identified (Fig. 1D, Supplementary Table S2). In the GSE67472 training set, GEO2R analysis identified 63 DEGs between normal and asthma groups, of which 39 were upregulated and 24 were downregulated (Supplementary Table S3, Fig. 1E). Intersecting the DEGs with CCC-related genes (Fig. 1F) and the DEGs with the yellow module genes (Fig. 1G) yielded 3 genes (Serpin Family B Member 2 (SERPINB2), Complement C3 (C3), and Complement C6 (C6)) and 4 genes (Colony Stimulating Factor 2 Receptor Subunit Beta (CSF2RB), Death Associated Protein Kinase 1 (DAPK1), Solute Carrier Family 16 Member 6 (SLC16A6), and CD69 Molecule (CD69)), respectively.

### Model construction and validation

**Figure 2:**
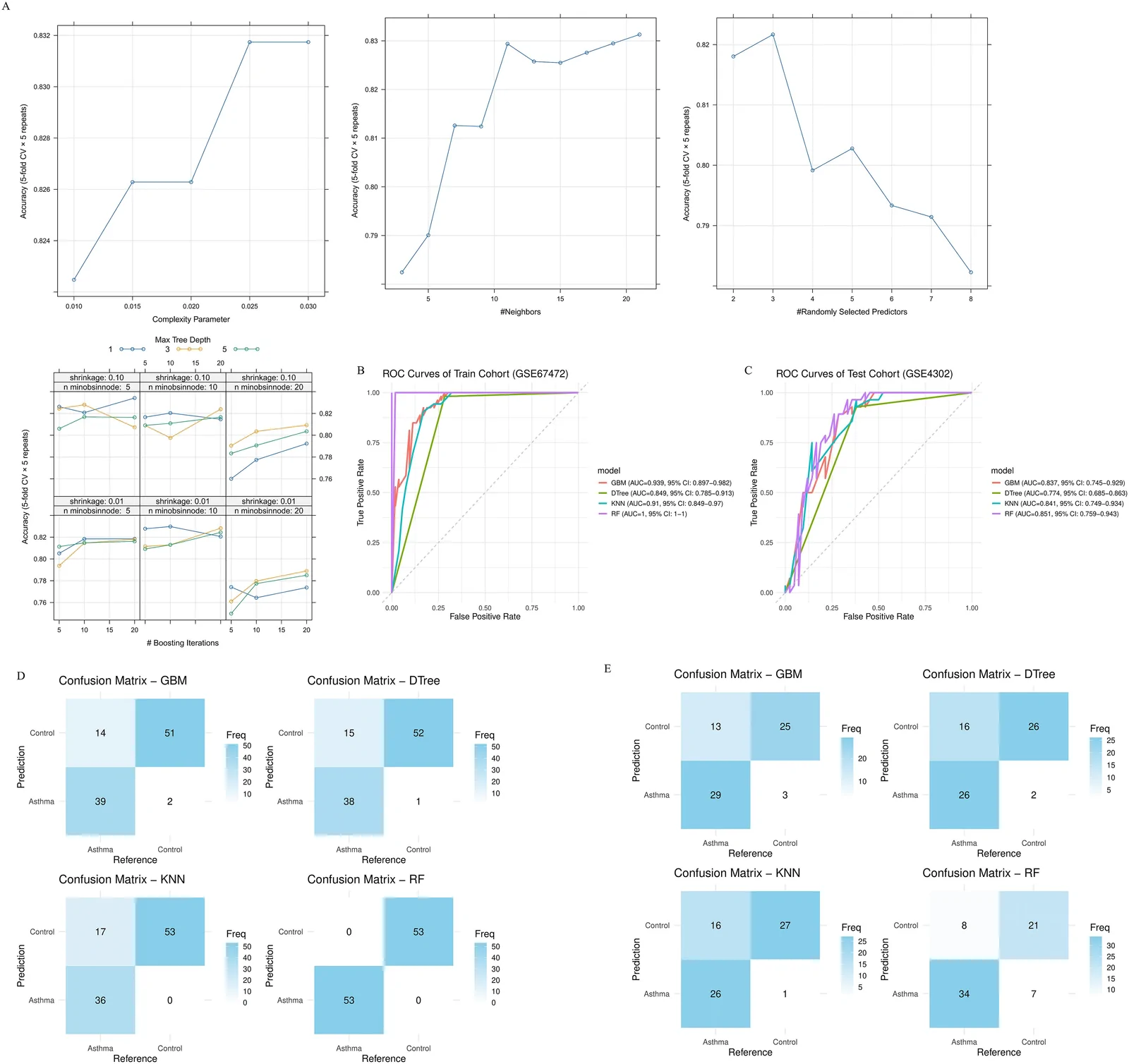
Construction and performance validation of asthma prediction models. (A) Comparison of accuracy for the four machine learning models—DTree, KNN, RF, and GBM—under different parameter settings (from left to right). Hyperparameter tuning curves of four machine learning models. The vertical axis represents the average classification accuracy derived from 25-fold validation (five-fold cross-validation repeated five times). Across the entire parameter tuning range, the ranges of SD of fold-wise accuracy for each model were as follows: DTree, 0.079–0.082; KNN, 0.042–0.060; RF, 0.074–0.080; and GBM, 0.061–0.092. (B, C) Model performance evaluation on the GSE67472 training set (B) and the GSE4302 validation set (C) using ROC curve analysis. (D–E) Confusion matrices of the four machine learning models in the GSE67472 training set (D) and independent GSE4302 validation set (E). Heatmaps visualize the counts of true positives (TP), false positives (FP), true negatives (TN), and false negatives (FN) for each model.

Based on the seven identified genes, four machine learning models—DTree, KNN, RF, and GBM—were constructed to predict asthma occurrence. The optimal hyperparameters for each model were determined as follows: the cp was 0.025 for DTree; the k was 21 for KNN; the mtry was 3 for RF; and for GBM, the n.trees was 20, the interaction.depth was 1, the shrinkage was 0.1, and the n.minobsinnode was 5 (Fig. 2A). On the GSE67472 training set, all four models demonstrated good discriminatory ability, with AUC values from their ROC curves exceeding 0.8 (Fig. 2B). In the independent validation set (GSE4302), the AUC values for all models remained above 0.75 (Fig. 2C). Five-fold cross-validation repeated five times was further performed to assess model stability, and the results are shown in Supplementary Fig. S1A. The mean cross-validation AUC of the RF model was 0.943 ± 0.045, with an ~0.09 difference from the AUC (0.851) of the independent external validation cohort. This difference fell within twice the SD of the cross-validation AUC, indicating favorable model stability and no obvious signs of overfitting. Among them, the RF model achieved the highest AUC. In order to further assess the clinical utility of the model, performance metrics including accuracy, F1-score, specificity, and sensitivity (recall) were calculated based on confusion matrices. The results indicated that the RF model performed best, achieving 100% in accuracy, precision, and recall. The GBM and DTree models showed comparable accuracy, while KNN had the highest recall but a lower precision (Fig. 2D and E). In summary, the RF model demonstrated the best overall performance among the constructed asthma prediction models.

### Identification of potential diagnostic biomarkers

**Figure 3:**
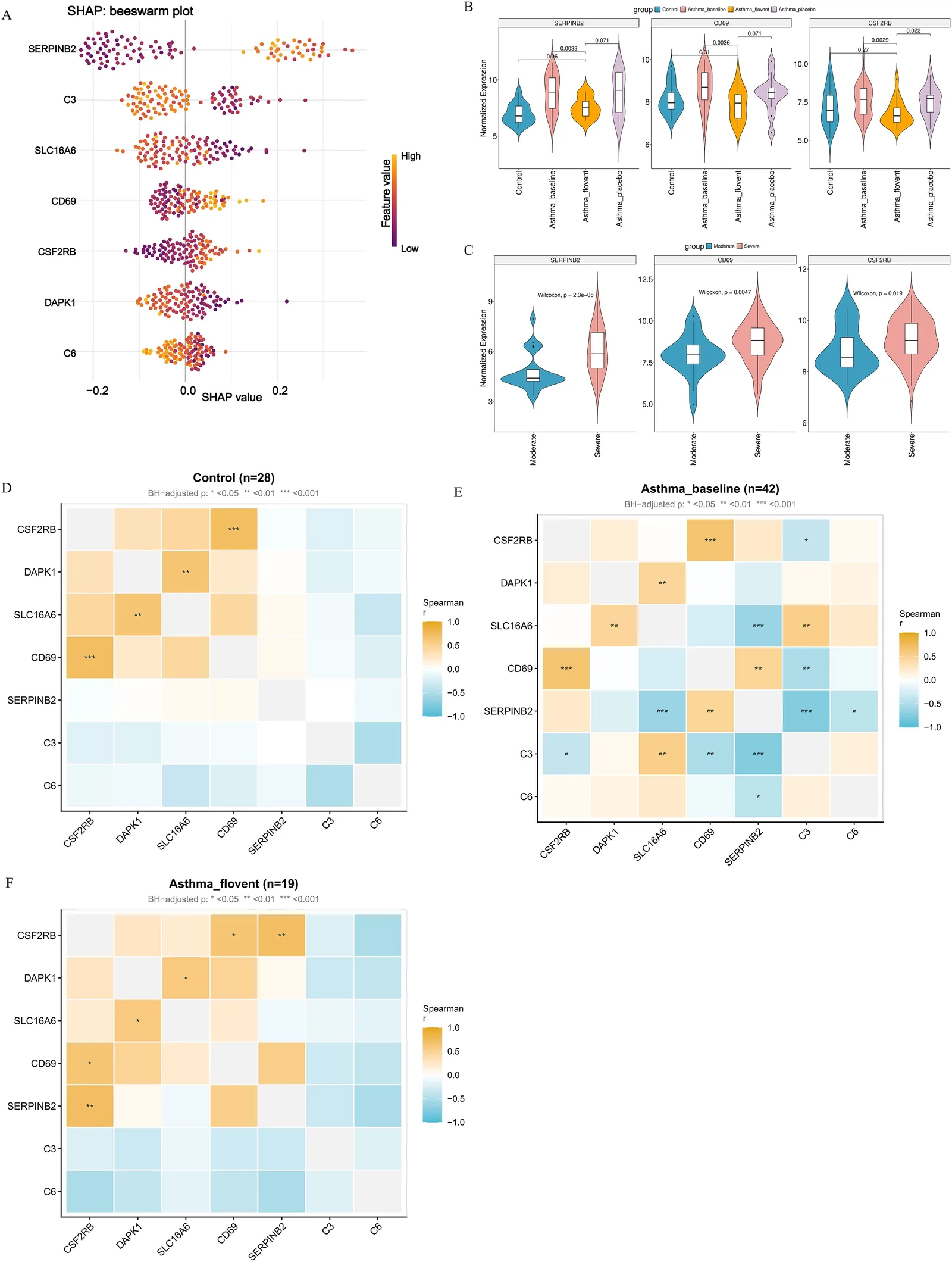
Identification of potential diagnostic biomarkers for asthma. (A) Feature importance ranking based on SHAP analysis, depicting the contribution of each feature to the RF model’s prediction of asthma. (B) Expression levels of SERPINB2, CD69, and CSF2RB across healthy control group, untreated asthma baseline group, Flovent treatment group, and placebo group in the GSE4302 dataset. Intergroup comparisons were performed, with corresponding *P* values labeled in the plot. (C) Differential expression of SERPINB2, CD69, and CSF2RB between patients with moderate and severe asthma in the GSE76262 dataset. The median (Q1–Q3) expression values were as follows: SERPINB2, 4.40 (4.21–4.92) for moderate asthma and 5.86 (5.00–7.15) for severe asthma; CD69, 7.96 (7.41–8.55) and 8.83 (7.93–9.57); CSF2RB, 8.54 (8.18–9.34) and 9.22 (8.70–9.89). (D–F) Spearman correlation heatmaps of hub genes in normal subjects (D), asthmatic patients before treatment (E), and asthmatic patients after treatment (F). *P* values were adjusted *via* the Benjamini–Hochberg method. ^*^, ^**^, and ^***^ denote adjusted *P* < .05, *P* < .01, and *P* < .001, respectively.

**Figure 4:**
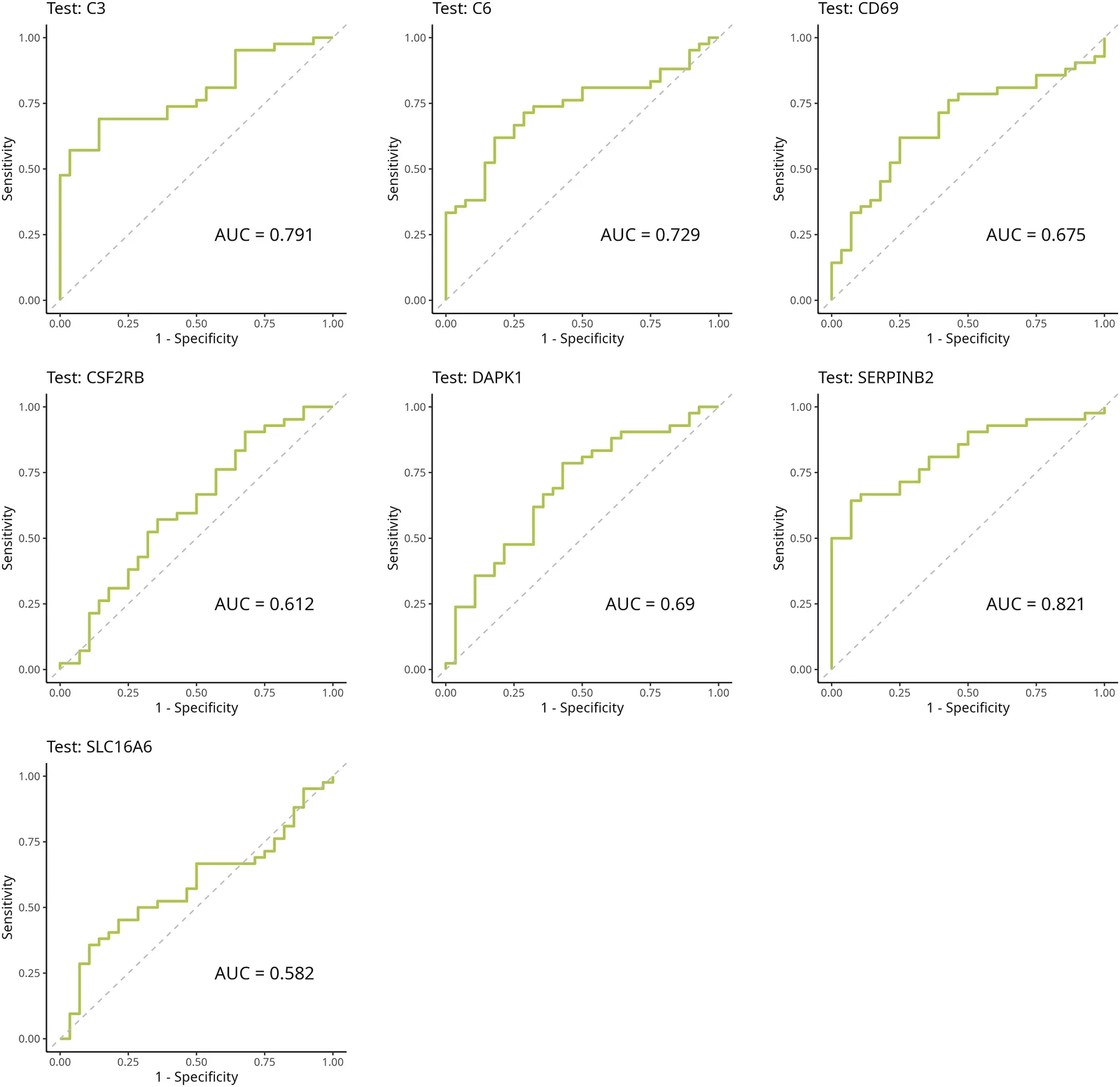
Performance evaluation of asthma diagnostic biomarkers based on ROC curve analysis. The ROC curves illustrate the performance of the seven genes—C3, C6, CD69, CSF2RB, DAPK1, SERPINB2, and SLC16A6—as diagnostic biomarkers in distinguishing asthma patients from healthy controls. The AUC is used to quantify the overall discriminatory efficacy of each biomarker.

SHAP analysis of the RF model revealed that the expression levels of the genes SERPINB2, CD69, and CSF2RB were positively correlated with the predicted probability of asthma, indicating their high contribution to the model’s asthma classification (Fig. 3A). Expression analysis showed that all three genes were highly expressed in asthma patients (Fig. 3B), consistent with previous findings [24–26]. Notably, the expression of these genes was markedly higher in patients with severe asthma compared to those with moderate asthma. Furthermore, their expression levels significantly decreased to near-normal levels following treatment with Flovent, while no such change was observed in the placebo group (*P* < .05, Fig. 3B and C). This indicates that the expression of these genes is closely linked to asthma severity and therapeutic response. Additionally, correlation analysis of the diagnostic genes across different groups (Fig. 3D−F) showed no significant correlation between C3 and SERPINB2 in normal samples or treated asthma samples, but a moderate and statistically significant negative correlation was observed in the untreated asthma group. A previous study has reported marked changes in SERPINB2 expression when C3 is inhibited (or when CD14 and C3 are co-inhibited) [27]. Moreover, the SHAP analysis revealed that both C3 and SERPINB2 had high contribution scores in distinguishing asthma phenotypes and exhibited the best single-gene diagnostic performance (highest AUC) (Fig. 4). These results suggest that C3 and SERPINB2 may have potential functional crosstalk during the initiation and progression of asthma. Nevertheless, current evidence is mainly derived from expression correlations and insufficient to confirm a direct regulatory interaction between the two molecules; further experimental validation is required to clarify their precise mechanisms.

To further evaluate the network interactions between candidate genes and the CCC pathway, correlation analyses were conducted on the 7 candidate genes and 80 detectable CCC pathway genes (Supplementary Fig. S1B). The analyses revealed that DAPK1 was significantly correlated with 29 CCC genes, including complement receptors CR1 (*r* = 0.484), C3AR1 (*r* = 0.430), C5AR1 (*r* = 0.410), complement components C1QA/B/C (*r* = 0.42–0.46), as well as complement regulatory proteins Complement Factor D (CFD) (*r* = 0.355) and Thrombomodulin (THBD) (*r* = 0.431). These co-expression relationships indicate that DAPK1 is tightly linked to complement activation and inflammatory cell recruitment in airway epithelium. Biologically, DAPK1-mediated apoptosis and autophagy in airway epithelial cells can trigger complement-dependent inflammatory cascades. Accumulating evidence has demonstrated that DAPK1 modulates the expression of complement components under inflammatory conditions *via* regulating the Nuclear Factor Kappa B (NF-κB) signaling pathway. SLC16A6 also exhibited significant correlations with 29 CCC genes, such as C5AR1 (*r* = 0.572), THBD (*r* = 0.508), CR1 (*r* = 0.501), C1QA/B/C (*r* = 0.43–0.48), C3 (*r* = 0.434), and SERPING1 (*r* = 0.394). SLC16A6 encodes monocarboxylate transporters that participate in lactate and ketone body transport. Its co-expression with complement genes may reflect the regulation of the metabolism-immune axis: inflammatory responses induced by complement activation elevate cellular metabolic demands, leading to upregulation of monocarboxylate transporters to meet energy requirements of activated immune and epithelial cells. In addition, lactate has been proven to modulate complement receptor signaling and macrophage polarization within the inflammatory microenvironment. Moreover, C3, C6, SERPINB2, CSF2RB, and CD69 were each significantly correlated with a variety of complement components, complement receptors, and coagulation regulatory genes. Among them, SERPINB2 mainly showed negative correlations, whereas CSF2RB and CD69 generally displayed extensive positive correlation patterns. Collectively, these findings imply that all seven candidate genes may participate in CCC-related biological processes through distinct co-expression profiles.

### Clinical expression validation of SERPINB2 and its functional roles in airway epithelial remodeling

**Figure 5:**
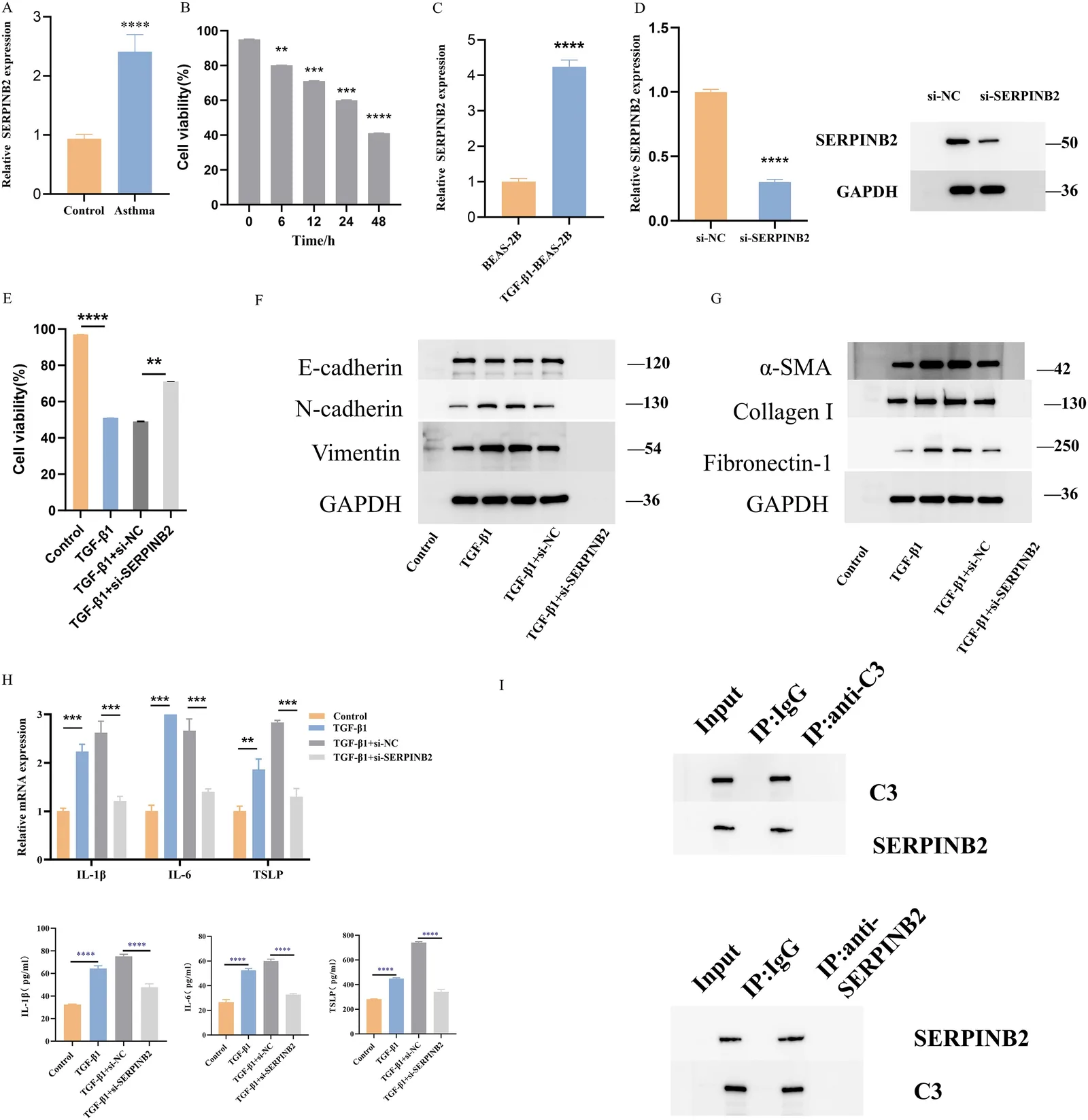
Clinical expression validation of SERPINB2 and its functional roles in airway epithelial remodeling. (A) qRT-PCR detection of SERPINB2 expression levels in tissues from healthy controls and asthma patients. (B) Viability of BEAS-2B cells treated with 10 ng/ml TGF-β1 for varying durations. (C) mRNA expression level of SERPINB2 in TGF-β1-stimulated cells assessed by qRT-PCR. (D) BEAS-2B cells were transfected with control siRNA (si-NC) or si-SERPINB2. After 48 h, cells were treated with 10 ng/ml TGF-β1 for an additional 48 h. SERPINB2 mRNA and protein expression post-transfection were detected by qRT-PCR and western blot, respectively. (E) Proliferation rate of TGF-β1-induced BEAS-2B cells with or without transfection. (F) Expression of EMT-related proteins E-cadherin, N-cadherin, and Vimentin. (G) Expression of airway remodeling-related proteins α-SMA, Collagen I, and Fibronectin-1. (H) mRNA expression levels of IL-1β, IL-6, and TSLP measured by qRT-PCR (upper panel), and corresponding protein concentrations in cell culture supernatants detected *via* ELISA (lower panel). (I) Co-IP assay was performed to detect the protein-level association between C3 and SERPINB2 in BEAS-2B cells. Input represents total protein sample control, and IgG serves as the negative control. ^**^P < .01; ^***^P < .001; and ^****^P < .0001.

To preliminarily validate the clinical expression profile of SERPINB2, qRT-PCR assays were performed on bronchial tissues collected from five asthma patients and five non-asthmatic controls. Compared with control tissues, SERPINB2 expression was markedly upregulated in asthmatic tissues (*P* < .01, Fig. 5A), providing preliminary clinical expression evidence supporting its potential as a candidate diagnostic biomarker. Subsequently, BEAS-2B cells were stimulated with TGF-β1 to construct an *in vitro* model recapitulating airway epithelial remodeling. The results showed that TGF-β1 treatment significantly reduced cell viability (*P* < .01, Fig. 5B) but increased the expression of SERPINB2 (*P* < .0001, Fig. 5C). Further gene silencing experiments mediated by siRNA demonstrated that transfection with si-SERPINB2 effectively reduced its mRNA and protein expression levels (*P* < .0001, Fig. 5D). Subsequent CCK-8 assays revealed that TGF-β1 treatment significantly inhibited cell viability, and this effect was reversed upon knockdown of SERPINB2 (*P* < .01, Fig. 5E). Given that epithelial−mesenchymal transition (EMT) has been reported to be associated with airway remodeling in asthma [28], this study also found that TGF-β1 stimulation led to decreased E-cadherin and increased N-cadherin and vimentin. Silencing SERPINB2 inhibited this TGF-β1-induced EMT process (Fig. 5F). Next, we examined the expression of proteins involved in airway remodeling [29] and typical cytokines [30–32] associated with asthma progression. The results indicated that TGF-β1 treatment markedly upregulated the expression of airway remodeling-related proteins (α-SMA, Collagen I, Fibronectin-1) and inflammatory cytokines (IL-1β, IL-6, and TSLP). Knockdown of SERPINB2 reduced the expression levels of these proteins in TGF-β1-treated cells (*P* < .01, Fig. 5G and H). Furthermore, Co-IP assays revealed an association between SERPINB2 and C3 at the protein level in BEAS-2B cells, suggesting that the two proteins may reside within the same protein complex. Nevertheless, this assay alone cannot confirm their direct binding interaction (Fig. 5I). The elevated expression of SERPINB2 in asthmatic tissues, together with its functional involvement in TGF-β1-induced phenotypes linked to airway epithelial remodeling, supports its potential as both a candidate diagnostic biomarker and a target for mechanistic investigations.

## Discussion

The CCC system participates in multiple chronic airway diseases by orchestrating innate immunity, inflammatory responses, and tissue repair. Its aberrant activation is closely linked to airway inflammation and structural remodeling in asthma [16, 33, 34], yet its precise mechanisms of action and clinical implications remain incompletely elucidated. Therefore, this research aimed to systematically identify key regulatory genes within the CCC pathway in asthma through an integrated bioinformatics analysis and to further explore their potential functional mechanisms. This work is expected to provide new perspectives and targets for the molecular subtyping and precise diagnosis of asthma.

This research employed WGCNA, along with four algorithms (DT, KNN, RF, and GBM), to screen genes and construct diagnostic models. Among them, the RF model demonstrated high diagnostic accuracy for asthma. SHAP analysis further revealed that the expression levels of three genes—SERPINB2, CD69, and CSF2RB—were positively correlated with the predicted probability of asthma, and their expression was markedly upregulated in asthma patients, particularly those with severe disease. Following treatment with Flovent, their expression levels markedly decreased to near-normal levels. This indicates a close association between the expression of these genes and asthma severity as well as treatment response. Existing research has confirmed that the SERPINB2 gene plays an important role in the development of asthma [35]. SERPINB2 can inhibit plasminogen activators, thereby preventing the activation of plasmin. Plasmin can directly degrade the ECM by removing glycoproteins or indirectly through the activation of metalloproteinases [17]. Consequently, inhibiting SERPINB2 to increase plasmin-mediated ECM turnover may reduce airway remodeling. SERPINB2 is recognized as an epithelial gene signature associated with Th2-type airway inflammation and can be induced by cytokines such as IL-13 [36–38]. In the present study, upregulated SERPINB2 expression was observed in clinical bronchial tissues and TGF-β1-treated BEAS-2B cells, further supporting its correlation with pathological airway processes in asthma.

Airway inflammation leads to damage and dysfunction of airway epithelial cells, thereby exacerbating the pathogenesis and progression of respiratory diseases [39, 40]. Previous studies have confirmed that TGF-β1 stimulation induces inflammatory responses in epithelial cells from asthma patients [41, 42]. In this research, treatment with TGF-β1 significantly increased the expression of IL-1β, IL-6, and TSLP. It is well-established that IL-1β, a pro-inflammatory cytokine with enhanced expression in asthma, has been reported as a potential drug target for asthma treatment [43, 44]. IL-6 is also a major inflammatory factor in asthma and can serve as a potential biomarker for disease severity [45, 46]. TSLP is a critical upstream regulator driving allergic inflammatory responses, and its importance in human asthma has been repeatedly demonstrated [47]. Experiments confirmed that knockdown of SERPINB2 inhibited the secretion of IL-1β, IL-6, and TSLP in TGF-β1-stimulated cells. Therefore, we conclude that SERPINB2 knockdown exerts an anti-inflammatory effect in asthma. Furthermore, airway inflammation not only elevates cytokine levels in bronchial epithelial cells but also impairs the integrity of the airway epithelium, thereby inducing airway remodeling and leading to irreversible damage to airway epithelial function [48]. Our experimental results show that TGF-β1 promotes the EMT process and increases the levels of airway remodeling-related proteins, while silencing the SERPINB2 gene inhibits this process. These findings indicate that SERPINB2 performs a key promotive role in airway inflammation and remodeling in asthma, positioning it as a potential therapeutic target.

As a core component of the complement system, C3 is activated under inflammatory conditions and hydrolyzed to generate active fragments such as C3a and C3b [49]. Among these, C3a, as a potent anaphylatoxin, can bind to its receptor (C3aR) to promote the degranulation of mast cells and eosinophils, releasing mediators like histamine and leukotrienes. This directly triggers characteristic asthmatic pathological changes, including bronchoconstriction and increased mucus secretion [50, 51]. Additionally, C3a can further recruit and activate Th2, regulating the production of Th2-type cytokines such as IL-4, IL-5, and IL-13, thereby amplifying allergic inflammatory responses [51]. Genetic studies have also shown that specific variations in the complement C3 and its receptor genes are closely associated with asthma susceptibility and severity [52]. Correlation analysis revealed a moderate statistically significant negative transcriptional correlation between C3 and SERPINB2 in untreated asthma samples, indicating a potential interaction between these two molecules. Co-IP assays demonstrated an association between SERPINB2 and C3 at the protein level in BEAS-2B cells, implying that they may coexist within the same protein complex. Notably, these two findings are not inherently contradictory. Transcriptional correlations reflect coordinated shifts in mRNA expression across individual patient specimens, which are readily modulated by cellular composition, inflammatory status, and feedback regulatory loops. In contrast, Co-IP detects whether proteins reside in a shared complex under defined cellular conditions, and such protein interactions can be shaped by post-translational modifications and protein stability. Accordingly, a negative mRNA-level correlation does not rule out physical association at the protein level. Meanwhile, Co-IP alone cannot confirm direct physical binding of the two proteins. Further experiments including purified protein binding assays, domain mapping, and functional blocking assays are required to characterize their binding mode and corresponding biological outcomes.

We integrated WGCNA with four machine learning algorithms to identify potential diagnostic biomarkers associated with asthma within the CCC. Among them, the diagnostic model based on RF demonstrated good performance. We further explored the biological characteristics of SERPINB2 *via* clinical tissue specimens and *in vitro* experiments. Nevertheless, the present study has several limitations. First, the sample sizes of the included GEO cohorts were relatively limited, and the distribution of clinical characteristics including age, geographic region, and asthma subtypes may be unbalanced. Although repeated five-fold cross-validation and independent external validation revealed no obvious overfitting, the RF model achieved nearly perfect classification performance in the training set, which may lead to an overoptimistic estimation of its predictive efficacy. Nested cross-validation should be adopted in future research, and multi-center, prospective large-scale cohorts are required to evaluate the robustness and generalizability of this model. Second, only five asthma patients and five non-asthmatic controls were recruited for clinical tissue validation. These samples can merely provide preliminary evidence of aberrant SERPINB2 expression and are insufficient for a systematic evaluation of its diagnostic sensitivity and specificity. In addition, the single-gene AUC values of CSF2RB and SLC16A6 were 0.612 and 0.582 respectively, indicating limited independent discriminatory power. These two genes were incorporated primarily based on their module relevance in WGCNA and potential synergistic contributions within the multi-gene model; feature ablation and incremental performance analyses are needed to verify their additional predictive value. Moreover, SERPINB2 expression exhibited considerable interindividual heterogeneity among patients with varying asthma severity. Its utility as an auxiliary biomarker for disease severity remains to be assessed in larger cohorts with detailed clinical stratification. Third, only TGF-β1-stimulated BEAS-2B cells were used for functional experiments. This model predominantly recapitulates the EMT and remodeling of airway epithelium and cannot fully mimic primary airway epithelial cells or the intact Th2-skewed asthmatic microenvironment. Follow-up validation experiments should be performed in primary bronchial epithelial cells, alternative airway epithelial models, and under stimulation with cytokines such as IL-4 and IL-13. Finally, the associations of DAPK1 and SLC16A6 with the CCC pathway are supported solely by co-expression data. Co-IP results only demonstrate an association between SERPINB2 and C3 at the protein level or their coexistence within a shared protein complex and cannot confirm direct binding between the two proteins. In future studies, further evaluation is needed to assess the effects of SERPINB2 knockdown on C3 expression and C3a/C3b generation. Combined with purified protein binding assays, domain mapping, and functional blocking experiments, *in vivo* studies, the interaction pattern and biological significance of this relationship should be clarified.

## Conclusion

In this study, integrative WGCNA and machine learning analyses identified seven asthma-related candidate genes, including SERPINB2, C3, C6, CSF2RB, DAPK1, SLC16A6, and CD69, and established a random forest model with favorable discriminatory performance for asthma. Clinical bronchial tissue validation further demonstrated elevated SERPINB2 expression in patients with asthma. Functional experiments showed that SERPINB2 knockdown alleviated TGF-β1-induced airway epithelial inflammation, epithelial–mesenchymal transition, and remodeling-related phenotypes. Moreover, Co-IP assays indicated a protein-level association between SERPINB2 and complement C3. Collectively, these findings highlight the potential diagnostic and biological relevance of complement and coagulation cascade-related genes in asthma and suggest that SERPINB2 may represent an important candidate linking airway epithelial inflammation and remodeling with complement-related processes. This study provides a basis for further investigation of molecular biomarkers and potential therapeutic targets for asthma.

## Supplementary Material

Supplementary_Figure_S1_elag007

Supplementary_Table_S1_elag007

Supplementary_Table_S2_elag007

Supplementary_Table_S3_elag007

## Data Availability

All data analyzed in this study were retrieved from public databases. The datasets GSE67472, GSE4302, and GSE76262 were downloaded from the Gene Expression Omnibus repository of the National Center for Biotechnology Information (https://www.ncbi.nlm.nih.gov/geo/). The gene set related to CCC was derived from the hsa04610 pathway in the KEGG database (https://www.kegg.jp/pathway/hsa04610). No new public datasets were generated in this study, and all data supporting the findings of this article are available from the above databases as well as the supplementary materials of this manuscript.

## Notes

**Jingjing Feng** is the Head Nurse of the Respiratory Medicine Department, Shangyu People's Hospital of Shaoxing, Shaoxing, Zhejiang, China. Her research interests include chronic airway inflammation in asthma, airway remodeling, refractory asthma, and the efficacy and predictive biomarkers of biological targeted therapies.

**Chengfang Xie** is an Associate Chief Physician in the Department of Respiratory Medicine, Shangyu People's Hospital of Shaoxing, Shaoxing, Zhejiang, China. Her research interests include pulmonary emphysema, small airway remodeling, and biomarkers for acute exacerbation of chronic obstructive pulmonary disease (AECOPD).

**Weiduo Jin** is an Attending Physician in the Department of Respiratory Medicine, Shangyu People's Hospital of Shaoxing, Shaoxing, Zhejiang, China. Her research interests include chronic airway inflammation in asthma, airway remodeling, interstitial lung disease, and pulmonary fibrosis.

**Yanhong Huang** is an Attending Physician in the Department of Respiratory Medicine, Shangyu People's Hospital of Shaoxing, Shaoxing, Zhejiang, China. Her research interests include chronic airway inflammation in asthma, airway remodeling.

**Yan Wang** is an Attending Physician in the Department of Neurosurgery, Nanjing Drum Tower Hospital, Affiliated Hospital of Medical School, Nanjing University, Nanjing, Jiangsu, China. Her research interests include hemorrhagic cerebrovascular diseases, such as intracranial aneurysms, cerebral arteriovenous malformations, dural arteriovenous fistulas, and cavernous hemangiomas; prediction of rupture risk.
